# Supplementary material for: Prevalence and associated factors of overweight/obesity among severely ill psychiatric patients in Eastern Ethiopia: A comparative cross-sectional study
Source: PLoS One. 2022 Mar 2;17(3):e0264461. doi: 10.1371/journal.pone.0264461 (PMC8890638; doi:10.1371/journal.pone.0264461)
Supplement: S1 File — (DOCX) [file pone.0264461.s001.docx]

**DIRE DAWA UNIVERSITY, COLLEGE OF MEDICINE AND HEALTH SCIENCES**


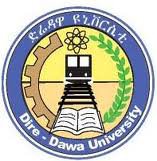


**PREVALENCE AND ASSOCIATED FACTORS OF OVERWEIGHT/OBESITY AMONG SEVERELY ILL PSYCHIATRIC PATIENTS IN EASTERN ETHIOPIA:**

**A COMPARATIVE CROSS-SECTIONAL STUDY**

BY:

1. DILNESSA FENTIE (MSc In MEDICAL PHYSIOLOGY)
2. TARIKU DERESE (MPH IN HUMAN NUTRITION)

A RESEARCH PROJECT TO BE SUBMITTED DIRE DAWA UNIVERSITY RESEARCH AFFAIRES DIRECTORATE

SEPTEMBER, 2021

DIRE DAWA, ETHIOPIA

**PREVALENCE AND ASSOCIATED FACTORS OF OVERWEIGHT/OBESITY AMONG SEVERELY ILL PSYCHIATRIC PATIENTS IN EASTERN ETHIOPIA: COMPARATIVE CROSSECTIONAL STUDY**

**PRINCIPAL INVESTIGATOR**

**DILNESSA FENTIE (MSc, IN MEDICAL PHYSIOLOGY)**

**CO-INVESTIGATORS:**

TARIKU DERESE (MPH, IN HUMAN NUTRITION)

SEPTEMBER, 2021

DIRE DAWA, ETHIOPIA

**ANNEX-IV. DECLARATION BY AUTHOR(S)**

We declare that this research report is a product of our effort and has not been presented to Dire Dawa University or any other institution in the same or different form for any purpose other than that for which we are now engagen and that all sources used for the research report we used have been duly acknowledged.

Principal Investigator:

Name Signature Date

Dilnessa Fentie ______________ _________

Co-Researcher (s):

Name Signature Date

Tariku Derese __________ ______________

# **Abstract**

***Background:*** Globally, the burden of overweight and obesity is a major cardiovascular disease risk factor and is even higher among patients with psychiatric disorders compared to the general population. This is mainly due to the deleterious lifestyles characterized by physical inactivity, excessive substance use, and unhealthy diets common among patients with psychiatric disorders, as well as the negative metabolic effects of psychotropic medications. Despite these conditions being a high burden among patients with psychiatric illness, little attention is given to them during routine reviews in psychiatric clinics in most African nations, including Ethiopia. Therefore, this study aimed to estimate and compare the prevalence of and associated risk factors for overweight and obesity among patients with psychiatric illnesses.

Methods: a comparative cross-sectional study was conducted between severely ill psychiatric patients and non-psychiatric patients in Dire Dawa, Eastern Ethiopia. The study included 192 study participants (96 psychiatric patients and 96 non-psychiatric controls). Weight and height were measured for 192 study participants. Baseline demographic and clinical characteristics of psychiatric and non-psychiatric patients were described. The data were cleaned and analyzed using the Statistical Package for Social Sciences, Version 21. The intergroup comparisons were performed using an independent sample t-test and Chi-square tests. Logistic regression analysis was used to identify the association between overweight/obesity and the associated variables.

Results: The magnitude of overweight/obesity was significantly higher in the severely ill psychiatric groups (43.8%) than in the non-exposed controls (20.80%). The prevalence of overweight/obesity was highest in major depressive disorders (40%), followed by schizophrenia (32%), and bipolar disorder (28%).

Conclusions: There was a high prevalence of obesity/overweight among psychiatric patients. Educational status, unemployment, and late stages of the disease were significant predictors of overweight/ obesity. Clinicians should be aware of the health consequences of overweight/obesity, and considering screening strategies as a part of routine psychiatric care is strongly recommended**.**

***KEYWORDS****:* Overweight; Obesity; major depressive disorder; non-psychiatric patients; Dire Dawa

# **Acknowledgment**

First and foremost, our deepest blessing goes Almighty of GOD for his generosity of
our healthy and wisdom to finish this project. As well, our sincere credit energies to study contributors for their participation, data collectors and supervisors for their assurance and effort throughout the long data collection period to keep up the data quality and realize this work and all my colleagues for their mental support. Last but not least, our gratitude goes to Dire Dawa University for its support and providing ethical clearance.

# **Table of Contents**

Contents

[**Abstract** ii](#_Toc89535478)

[**Acknowledgment** iii](#_Toc89535479)

[**Table of Contents** iv](#_Toc89535480)

[**List of Figures** vi](#_Toc89535481)

[**List of Tables** vii](#_Toc89535482)

[**List of Abbreviation** viii](#_Toc89535483)

[**1.** **Introduction** 1](#_Toc89535484)

[**1.1.** **Significance of Study** 2](#_Toc89535485)

[**1.2.** **Conceptual Framework** 3](#_Toc89535486)

[**2. Objectives of Study** 5](#_Toc89535487)

[**3.1. General Objective** 5](#_Toc89535488)

[**3.2. Specific Objectives** 5](#_Toc89535489)

[3. **Materials and Methods** 6](#_Toc89535490)

[**4.2** **Study setting and Period** 6](#_Toc89535491)

[**4.3** **Study design** 6](#_Toc89535492)

[**4.4** **Source Population** 6](#_Toc89535493)

[**4.5** **Study Population** 6](#_Toc89535494)

[**4.6** **Selection Criteria** 6](#_Toc89535495)

[**4.6.1** **Inclusion criteria** 6](#_Toc89535496)

[**4.6.2 Exclusion Criteria** 7](#_Toc89535497)

[**4.7. Sample size estimation and Sampling Technique** 7](#_Toc89535499)

[**4.8. Study Variables** 7](#_Toc89535500)

[4.9. **Operational Definition/Standard definition** 7](#_Toc89535501)

[**4.10. Data Collection tools and Procedures** 8](#_Toc89535502)

[. **4.10. 1. Anthropometric** 8](#_Toc89535503)

[**4.12. Data Analysis and presentation** 9](#_Toc89535504)

[**4.13. Ethical Consideration** 9](#_Toc89535505)

[4. Results 10](#_Toc89535506)

[5.1 Socio- Demographic Characteristics of the study groups 10](#_Toc89535507)

[**5.2. The Prevalence of Overweight/Obesity** 13](#_Toc89535508)

[5.3. **Overweight/obesity patterns in various psychiatric disorders** 14](#_Toc89535512)

[**6. Discussion** 18](#_Toc89535514)

[**Limitations of the Study** 19](#_Toc89535515)

[7. Conclusion 19](#_Toc89535516)

[**8. Recommendations** 20](#_Toc89535517)

[**7. References** 21](#_Toc89535518)

[**8. Annex** 25](#_Toc89535519)

# **List of Figures**

[Figure 1:Conceptual frame work developed after revising different literatures factors that affect overweight/obesity status of patient with psychiatric illness](#_Toc51230639) [4](#_Toc89535635)

[*Fig 2: Shows the prevalence of overweight/obesity among psychiatrically exposed groups and non-psychiatric controls in Dire Dawa, Eastern Ethiopia, 2021.* 13](#_Toc89535637)

[*Fig 3: The Burden of Overweight/ Obesity Among Different Psychiatric Disorders Dire Dawa, Eastern Ethiopia, 2021* 14](#_Toc89535639)

# **List of Tables**

[Table 1 Characteristics of psychiatric(exposed) and non-psychiatric patients (non-exposed) at Eastern,Eastern Ethiopia, 2021. 19](#_Toc79243228)

[Table 2. The magnitude of overweight/obesity among psychiatric and non-psychiatric study participants at Eastern Ethiopia, 2021 21](#_Toc79243229)

[Table 3 Bivariate logistic regression analysis associated factors of overweight/obesity among severely ill psychiatric patients at Eastern Ethiopia,2021 22](#_Toc79243230)

[Table 4 multivariate logistic regression analysis associated factors of overweight/obesity among severely ill psychiatric patients at Eastern Ethiopia,2021 24](#_Toc79243231)

# **List of Abbreviation**

AOR- Adjusted odds ratio

BMI- Body mass index

COR Crude odds ratio

WHO- World Health Organization

# **Introduction**

Obesity/overweight have reached an epidemic burden globally, with at least 2.8 million people dying each year as a result of being overweight or obese[1]. According to World Health Organization (WHO) European Region, an estimated 23% of women and 20% of men are obese or overweight[2]. The magnitude of overweight and obesity among adults in Africa is 27% and 8% respectively [3]and the report from the Ethiopia Demographic and Health Survey(EDHS) the burden of overweight/obesity is 8 %[4]. Overweight and obesity are major risk factors for a number of chronic diseases, including diabetes, cardiovascular diseases, and cancer. Obesity is frequently accompanied by depression, and the two can trigger and influence each other. Obesity and overweight are major public health issues, as well as the leading preventable cause of death in both developed and developing countries[5].

A study has shown that the burden of obesity reveals that more than 1.9 billion adults aged 18 and older were overweight in 2014; over 600 million were obese; 39% of adults aged 18 and over were overweight, and 13% were obese[6]. Overweight and obesity are on the rise worldwide, not only in the general population but also in psychiatric patients. The major causes of overweight and obesity are not clear but are considered multi-disciplinary and related to genetic, metabolic, and psychological factors[7,8].

The relationship between abnormal body weight and psychiatric disorders has continuous, complex relations that are still being debated. Some scholars suggest that overweight/obesity may cause common psychiatric disorders, whereas others have found that psychiatric patients are more prone to obesity[9,10]. A community-based study of Saudi Arabian university students showed that obesity and overweight were positively associated with several mental disorders, especially mood disorders and anxiety disorders[11]. Another study done in Egypt showed that the prevalence of obesity and overweight in psychiatric patients was 66.93% (22.31% were obese, and 44.62% were overweight). The prevalence of obesity was highest in bipolar disorder (41.38%), followed by depression (37.93%), schizophrenia (10.34%), anxiety disorder (6.9%), and finally substance abuse disorder (3.45%), but the difference was not statistically significant. There was a significant correlation between the sociodemographic characteristics of patients with obesity and the distribution of psychiatric disorders[12].

In developing countries, along with economic development and income growth, the number of people who are overweight or obese is increasing. Among the reasons for the increasing obesity in the population of poor people are higher unemployment, lower education level, irregular meals, and low physical activity, which among the poor is associated with a lack of money for sports equipment[13] . In spite of such a burden of overweight/obesity in the community, there is no available scientific data in our region, Ethiopia. Thus, the purpose of this study is to compare the prevalence of overweight/obesity in patients with severely ill psychiatric patients at an Eastern Ethiopia psychiatric center as compared to non-exposed controls. The authors hypothesized that the prevalence would be higher in patients with psychiatric disorders. This study also determined the associated factors of overweight/obesity.

## **Significance of the Study**

There are limited studies done regarding to overweight/obesity among psychiatric patient in Ethiopia and in other sub-Saharan Africa countries. This study is intended primarily to assess the magnitude of overweight/obesity and link of psychiatric disorder with overweight/obesity and determinants factors of overweight/obesity. So, this study output might be to push health care professionals to promote awareness on risky behavioral factors, strengthen early detection and treatment of components of overweight/obesity to reduce premature mortality in patients with psychiatric disorder.

The findings of this study will serve also as a benchmark for health planners to implement appropriate preventive and control measures, so as to alleviate the double burden of overweight/obesity patients with psychiatric disorder. This study will also give baseline information to other public and private organizations to wake up on screening and intervention schedule for psychiatric patients to reduce the impacts of chronic non-communicable diseases. It will also add additional knowledge to the existing literature so as to trigger different researchers to conduct further study on the link of psychiatric disorder on overweight/obesity at large.

- 1. **Conceptual Framework**

**Behavioral factors:**

-Cigarrete smoking

-Alcohol intake

-physical inactivity

**Clinical history**

-Types of psychiatric disorder

-Duration of mental illness

- Types of psychiatric medication

-Family history of HTN & DM

-

Overweight/Obesity

**Sociodemographic variables**

-Age, Sex, marital status,

Educational level, residency,

income per month, occupation

Figure 1**:** Conceptual frame work for overweight/obesity among patient with psychiatric disorders

# **2. Objectives of the Study**

## **3.1. General Objective**

The main aim of the study was to compare the magnitude of overweight/obesity in patients with severely ill psychiatric patients and non-psychiatric individuals and to verify associated factors of overweight/obesity at Eastern Ethiopia psychiatric center, Ethiopia, 2021.

## **3.2. Specific Objectives**

1. To estimate and compare the magnitude of overweight/obesity; patients with psychiatric illness and non-psychiatric controls
2. To evaluate associated factors of overweight/obesity among psychiatric patients.

# **Materials and Methods**

## **Study setting and Period**

The study was conducted at Eastern Ethiopia psychiatric center from January 5 to June 10,2021. The hospital is located in 515 kilometers away from the capital city, Addis Ababa, Ethiopia.. The psychiatry unit gives services through the outpatient and inpatient departments by a dedicated team of psychiatrist doctors, master’s psychiatric clinical officers, psychiatric nurses, and supportive staff. The psychiatry unit provides service for more than 4500 clients a year and 1250 clients registered for follow-up visit. Monthly and weekly patient flow in the clinic ranges from 390-420 and 110-160 respectively as observed during preliminary survey.

## **Study design**

Institutional based comparative crossectional study was employed between patients with psychiatric illness and non-psychiatric individuals.

## **Source Population**

All clients attending outpatient department for treatment or medical advice during the study period; were the source population.

## **Study Population**

All selected clients attending outpatient department for treatment or medical advice during the study period; were study population.

Severe psychiatric illness/ disorders (exposed/cases)- established diagnosis of a common psychiatric disorder include schizophrenia, schizoaffective disorder, major depressive, and bipolar disorders recruited to this study. The psychiatric diagnosis of subjects obtained from the patients’ records and relied on the Diagnostic and Statistical Manual of Mental Disorders (DSM-5).

Non-psychiatric study participants (non- exposed group/controls) will be age (±2 years) and sex matched individuals, consisted of volunteers who did not have any psychiatric diagnoses, and who attend outpatient department for general medical or surgical treatment other than psychiatric disorders, or who had no lifetime diagnosis or treatment for a mental illness.

## **Selection Criteria**

## **Inclusion criteria**

Both men and women, aged 18 and above who were stable (in remission phase or having recovered from an acute episode). All consented severely ill psychiatric patients who were attend the clinic during the study period and on psychiatric treatment were included to the study.

### **4.6.2 Exclusion Criteria**

## The current history of pregnancy and physical deformities were excluded from the study. Clients who were unstable or unable to consent (as determined by the attending physician) were also excluded

## **4.7. Sample size estimation and** **Sampling Technique**

The sample size was determined using the analytical study sample size calculation formula by taking a two-sided confidence level of 95%, a power of 80% with a double proportion formula and an equal number of cases to controls[15]. A total sample of 192 study participants (96 psychiatric patients and 96 non-psychiatric individuals) was endorsed after considering a 10% non-response rate[16,17]. All psychiatric patients were selected using consecutive sampling techniques. After collecting data from a single psychiatric patient, one corresponding age and sex-matched non-psychiatric control was chosen.

**4.8. Study Variables
 4.8.1 Dependent Variables**

Overweight/Obesity
 **4.8.2. Independent Variables**

Marital status, level of education, residence, occupation status body mass index, family history of (diabetes mellitus, hypertension), smoking, alcohol consumption, exercise habit, clinical characteristics (patterns of psychiatric disorders, duration of disease, and types of psychiatric medication), physical inactivity were categorized as independent variables.

## 4.9. **Operational Definition/Standard definition**

**Body mass Index (BMI)** was calculated as the weight of the individual in kilograms divided by height in meter square. With respect to the body mass index (BMI), there are four groupings: underweight (BMI < 18.5 Kg/m^2^), normal (BMI between 18.5 and 24.9.Kg/m^2^), overweight (BMI between 25 and 29.9 Kg/m^2^), and obese (BMI ≥ 30 Kg/m^2^)[18].

**Vigorous-intensity activity** was defined as any activity that results in a significant increase in breathing or heart rate if performed for at least 75 minutes per week. **Moderate-intensity activity** **was** defined as any activity that causes a small increase in breathing or heart rate if continued for at least 150 minutes per week or walking for at least 30 minutes per day. **Sedentary** involves a person not meeting any of the above-mentioned criteria for the moderate- or high-level categories[19].

**Smoking state:** Non-smoker or ever not smoked, which is coded as (0=no) and all smoker (current, current daily, and past smokers) which is coded as (1=yes) [20].

**Alcohol consumption**. Ever consumer / consumer of any alcohol represents current alcoholic drinker (past 30 days) which is coded as (0=No and 1=Yes) and past alcoholic drinker (drank in the past 12 months) which is coded as (0=No and 1=Yes) [21].

## **4.10.** **Data Collection tools and Procedures**

All the study groups were interviewed, and examined. The questionnaires were adapted from the WHO STEP wise approach recommended for non-communicable disease surveillance. The data were collected by psychiatrist doctor and trained psychiatric nurses with close supervision of the investigators. The data collectors applied the same procedures to both the psychiatric and non-psychiatric clients to collect data.

### . **4.10. 1. Anthropometric**

The height and weight of each subject were measured by using a scale to the nearest 1 cm and 1 kg, respectively. The height of a subject was measured by using an erect height measuring scale. Measurements of height were made with the subject's bare feet. The subjects stood straight against the erect measuring scale, and their heads, shoulders, buttocks, and heels touched the scale. The subject's axis of vision was horizontal. Then, they took a deep breath to relax their shoulders. With a flat object, the upper level of their heads was marked against the scale and measured to the nearest 1 cm. The weight of the study participants was measured using a calibrated weighing scale with the participant not wearing shoes and heavy clothes.

**4.11.** **Data quality control**

The data collecting tools prepared in English were translated to Amharic and retranslated back to English, to confirm the correctness of the translation. Two days training were given to the data collectors about the purpose of the study, measurement technique and ethical consideration. Mock interviews and practical field exercise were given to data collectors to ensure the quality of the field operation. All questionnaires were checked daily for completeness, accuracy and clarity by the investigators. Furthermore, the data were checked during entry and compilation before analysis. Pretest of data collection tool (questionnaire) was done by pre-testing 5% of questionnaires at Goro health center, and necessary corrections were made prior to the actual data collection period.

## **4.12.** **Data Analysis and presentation**

The data were cleaned then entered into Epi Data 3.1, then exported to Statistical Package for Social Sciences (SPSS), Version 20 for statistical analysis. Descriptive data analysis was done to check missing values, potential outliers and the distribution for those continuous variables. Descriptive of the results were reported using frequency (percentage) for categorical variables and mean ± SD (or median with interquartile range) for continuous measures respectively. The Chi-square test and Student t-test were used respectively for comparison of categorical and continuous variables between the two study groups. Logistic regression was used to analyze the factors associated with overweight/obesity. First, the association between each of the potential predictor variables and overweight were examined ignoring other variables by using bivariate analysis. A variable with p-value < 0.25 and variables considered as medically significant were a candidate for multivariable logistic regression. Odds ratios and 95% confidence intervals were calculated to determine the independent risk factors associated with overweight/obesity. Data were presented using narrative, figures and tables from the result of statistical analysis. p value <0.05 was deemed to be statistically significant.

## **4.13.** **Ethical Consideration**

Ethical clearance was obtained from the Institutional Review Board (IRB) of Dire Dawa University, college of medicine and health sciences (protocol no. 300/55//2021). The regional health office and hospital higher officials agreed to the carrying out the study. A letter of cooperation was obtained from Dire Dawa regional health office before the study commenced. A written informed consent was obtained from all study participants at the time of the study. Permission was asked to respondents and none of them was forced to participate in this study. Any identifiable issue was eliminated to ascertain confidentiality. All study participants who have overweight/obesity were referred to a respective physician for further evaluation.

. **4.14. Dissemination of the Result**

The research paper will be prepared in copies and summited to Dire Dawa regional health office and Dire Dawa University Research Directorate office. The result will be communicated with the stakeholders through presentations on meeting, scientific panels and workshops after approved by Dire Dawa University scientific community. Finally, to ministry of health and moreover the results will be sent for publication in reputable Journals.

# Results

5.1 Socio- Demographic Characteristics of the study groups

A total of 192 study participants (96 cases and 96 controls) participated in the study to determine the burden of overweight or obesity among severely ill psychiatric patients. The mean age (years) of psychiatric patients was 37.18 ±12.59 and 36.59±13.56 in the non-exposed group, which was a statically insignificant difference (p = 0.754). Around 32.2% (31) of the exposed patients and 44.8% (43) of the non-exposed study participants attended college and above educational level (Table 1).

*Table 1: Sociodemographic characteristics of psychiatric and non-psychiatric controls, Dire Dawa, Eastern Ethiopia 2021*

| Characteristics | | Cases(psychiatric patients) | Controls | *p* value |
| --- | --- | --- | --- | --- |
| Sex | .561* | | | |
| Male | | 55 (57.3%) | 56(58.3%) |  |
| Female | | 41(42.7%) | 40(41.7%) |  |
| Age in  years (mean±SD) | 37.18±12.59 36.59±13.56 0.754** | | | |
| Educational status | .076* | | | |
| No formal education | | 45(46.9%) | 26(27.1%) |  |
| Primary education | | 3(-) | 6(6.3%) |  |
| Secondary education | | 17(17.7%) | 21(21.9%) |  |
| College & above | | 31(32.2%) | 43(44.8%) |  |
| Marital status | **0.743*** | | | |
| Single/unmarried | | 35(36.5%) | 33(34.4%) |  |
| married | | 12(12.5%) | 33(34.4%) |  |
| Divorced/separated/died | | 12(12.5%) | 13(13.5%) |  |
| Residence | **0.009*** | | | |
| Rural | | 59 (61.5%) | 41(42.7%) |  |
| Urban | | 37(38.5%) | 55 (57.3%) |  |
| Employment | **0.061*** | | | |
| Unemployed | | 72(75.0%) | 36(37.5%) |  |
| Employed | | 24(25.0%) | 60(62.5%) |  |
| Ever smoked | |  |  | 0.86* |
| No | | 79 (82.3%) | 82 (85.4%) |  |
| Yes | | 17 (17.7%) |  |  |
| Ever Alcohol Intake | |  |  | .401* |
| No | | 70(72.9%) | 75 (78.1%) |  |
| Yes | | 26(27.1%) | 21 (21.9%) |  |
| physical activity | **0.521*** | | | |
| Yes | | 39(40.6%) | 29(30.2%) |  |
| No | | 57(59.4%) | 67(69.8%) |  |
| Family history of hypertension | **0.082*** | | | |
| Yes | | 36(37.5%) | 13(13.5%) |  |
| No | | 69(71.9%) | 83(86.5%) |  |
| Family history of diabetes | **0.013*** | | | |
| Yes | | 27(28.1%) | 13(13.5%) |  |
| No | | 68(70.8%) | 83(86.5%) |  |
| *SD=standard deviation*, Independent sample t test, *Pearson’s chi square test |  | | | |

# **5.2.** **The Prevalence of Overweight/Obesity**

The prevalence of overweight/obesity was significantly higher among the severely ill psychiatric groups than the in non-exposed controls (p = 0.001). The prevalence of overweight/obesity among psychiatric patients was 43.8% (95% CI: 33.3-55.3%), whereas the magnitude of overweight/obesity among non-psychiatric controls was 20.80% (95% CI: 16.5-28.9%) (Fig 1).


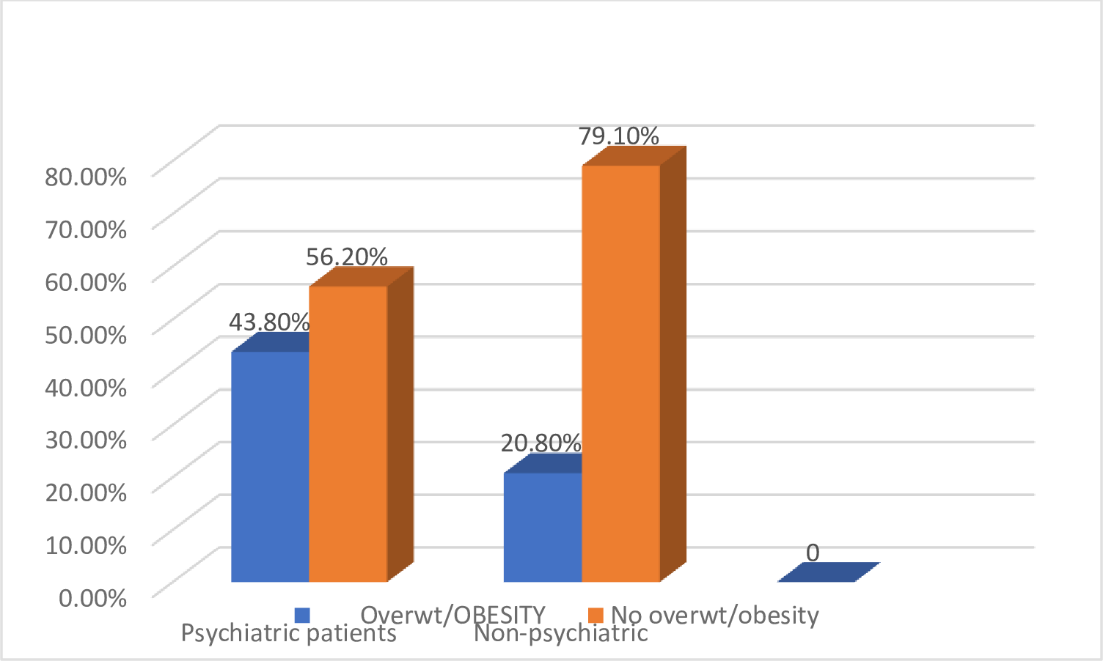


*Fig 1: Shows the prevalence of overweight/obesity among psychiatrically exposed groups and non-psychiatric controls in Dire Dawa, Eastern Ethiopia, 2021.*

5.3. **Overweight/obesity patterns in various psychiatric disorders**

The prevalence of overweight/obesity was highest in 40% of patients with major depressive disorder (MDD), followed by schizophrenia 32%, bipolar disorder, and 28%. There was a significant correlation between overweight/obesity and the distribution of psychiatric illnesses (Fig 2).

*Fig 2: The Burden of Overweight/ Obesity Among Different Psychiatric Disorders Dire Dawa, Eastern Ethiopia, 2021*

**5.4.** **Factors associated with overweight or obesity**

All covariates that with a p-value ≤0.2 in the bivariate analysis were included in the multivariate analysis. In this study age, sex, marital status, educational level, physical activity level, occupational status, and duration of psychiatric illness were significantly associated with overweight or obesity status in the bivariate analysis. After adjusting variables such as study participants who were uneducated (AOR = 2.2, 95% CI (1.57, 8.5), p = 0.014), unemployed (AOR = 6.5, 95% CI (1.8, 35.20), p =0.023), and duration of psychiatric illness (AOR = 2.9, 95% CI (1.23, 4.1), p = 0.02), were independent predictors of overweight/obesity(Table 3

*Table 3: Factors associated to Overweight/ Obesity among severely ill psychiatric patients, Dire Dawa, Eastern Ethiopia, 2021*

| Variables | Category | Overweight/Obesity  Yes No | | COR (95%CI) | AOR (95%CI) |
| --- | --- | --- | --- | --- | --- |
| Age (years) | 18-30 | 8(26.7%) | 22(73.3%) | 1 | 1 |
|  | 31-40 | 12(46.2%) | 14(53.8%) | 2.3(.77,7.20) | 1.4(.33,5.9) |
|  | 41-50 | 13(61.9%) | 8(38.1%) | 4.4(1.35,14.7) | 1.6(.3, 7.9) |
|  | >50 | 9(47.4%) | 10(52.6%) | 2.4(.73,8.3) | .71(.13,3.7) |
| Gender | Male | 20(38.5%) | 32(61.5%) | 1 | 1 |
|  | Female | 22(50.0%) | 22(50.0%) | 1.6(.70,3.60) | 1.3(.53,5.02) |
| Marital status | Single | 12(34.3%) | 23(65.7%) | 1 | 1 |
|  | Married | 23(46.9%) | 26(53.1%) | 1.6(.69,4.1) | 2.3(.75,7.41) |
|  | Widowed | 7(58.3%) | 5(41.7%) | 2.6(.70,10.2) | 2.8(.42,18.8) |
| Education  status | Uneducated | 25(55.6%) | 20(44.4%) | 3.6(3.13,18.9) | 2.2(1.57,8.5) |
|  | primary | 2(66.7%) | 1(33.3%) | .33(.10,1.1) | 7.7(.16,8.9) |
|  | secondary | 5(29.4%) | 12(70.6%) | .38(.14,.99) | 1.1(.19,6.9) |
|  | college and above | 10(32.3%) | 21(67.7%) | 1 | 1 |
| Occupation | employed | 3(12.5%) | 21(87.5%) | 1 | 1 |
|  | Non employed | 39(54.2%) | 33(45.8%) | 8.2(2.26,30.2) | 6.5(1.8,35.20) |
| Residency | rural | 20(33.9%) | 39(66.1%) | 1 | 1 |
|  | urban | 22(59.5%) | 15(40.5%) | 2.8(1.22,6.6) | 1.3(.38,4.5) |
| Smoking | yes | 11(64.7%) | 6(35.3%) | 2.8(.95,8.4) | 2.9(.6, 13.5) |
|  | No | 31(39.2%) | 48(60.8%) | 1 | 1 |
| Alcohol intake | yes | 13(50.0%) | 13(50.0%) | 1.4(.5, 3.4) | 1.01(.25,4.01) |
|  | No | 29(41.4%) | 41(58.6%) | 1 | 1 |
| physical exercise | Yes | 14(35.9%) | 25(64.1%) | 1 | 1 |
|  | No | 28(49.1%) | 29(50.9%) | 1.7(.74,3.9) | 1.2(.41,3.7) |
| Duration of psychiatric illness  (years) | <5 | 10(40.0%) | 15(60.0%) | 1 | 1 |
|  | 5-10 | 7(31.8%) | 15(68.2%) | .45(.04,4.2) | 1.3(.28,6.1) |
|  | >10 | 25(51.0%) | 24(49.0%) | 3.58(2.10,3.18) | 2.9(1.23,4.1) |

BMI=Body mass index, COR=crude odd ratio, AOR=adjusted odd ratio

**6****. Discussion**

To the best of our knowledge, no previous study has been conducted in Ethiopia to determine the prevalence of excess weight among severely ill psychiatric patients. The results showed that the study groups (severely ill psychiatric patients and non-psychiatric controls) were similar in terms of age, sex, marital status, and educational level; with no statistically significant difference.

Our findings revealed a significantly higher prevalence of overweight/obesity (43.8%) among psychiatric patients than among non-psychiatric controls (20.8%), p<0.05. Thus, possibly due to the deleterious lifestyles characterized by physical inactivity, excessive substance use, and unhealthy diets common among patients with psychiatric disorders, they may be predisposed to overweight or obesity. This study is consistent with the findings reported from the United States, where patients with severe psychiatric disorders had a higher average BMI than the subjects in the general population controls(32.11, SD = 7.72vs 27.62, SD = 5.93,P = 0.000) in 2017 [22], and in Egypt by Ahmed kamel et al, in 2016[12]. However, the current study has a higher prevalence of overweight/obesity as compared to the WHO report in 2016[6] and by Mekonnen et al.in 2018[23]. This could be due to variations in study participants (in our study, the study participants were severely ill psychiatric patients) and lifestyle and physical activity differences.

Furthermore, the current study shows a higher prevalence of overweight/obesity among those with major depressive disorders (MDD) as compared to schizophrenia and bipolar disorders. This finding is supported by previous studies done in the Campania region by Micanti F, Pecoraro G, Mosca P, et al., 2017[25] and in McLean Hospital, Belmont, MA, USA by Chouinard, V.-A., et al.[24] and Francesco Weiss , Margherita et al.2020[26]. This is partly explained by depressed patients reduced physical activity, which results in positive energy balance and overweight/obesity disorders. Many people who have difficulty recovering from sudden or emotionally draining events unknowingly begin eating too much of the wrong foods or forgoing exercise[27]. Mechanisms underlying this weight gain include lifestyle and environmental factors and psychiatric medications, though emerging evidence has also suggested the role of genetic and neuroendocrine processes[28]. The current study also, found that education status, employment, and increased duration of psychiatric illness were significant predictor of obesity/overweight among psychiatric patients. The current study also found that education status, employment, and increased duration of psychiatric illness were significant predictors of obesity/overweight among psychiatric patients. This finding is consistent with the study conducted by Husky et al. in 2017[29,30].

**Limitations of the Study**

To the best of our knowledge, this is the first study in Ethiopia to provide information on the problem of overweight/obesity among the psychiatric population as compared to the non-psychiatric population. The study's shortcomings include the absence of dietary habits, social desirability, and the possibility that recall biases influenced the results.

7. Conclusion

The current study described the burden of overweight or obesity and associated factors among psychiatric patients compared to non-psychiatric controls at Dire Dawa psychiatric center, Ethiopia. The magnitudes of overweight and obesity were significantly higher among the severely ill psychiatric group (43.8%) than in non-exposed controls (20.80%). It was found that educational status, unemployment, and late stages of the disease were significant predictors of overweight or obesity. Clinicians should be aware of the health consequences of overweight and obesity and consider instituting targeted weight treatment programs as a part of routine psychiatric care. This is strongly recommended. The current study also found that education status, non-employment, and the late stage of psychiatric diseases were significant predictors of obesity and overweight. Physicians should be aware of the health consequences of obesity and should consider instituting targeted weight treatment programs as a part of routine psychiatric care.

# **8. Recommendations**

The present study has disclosed the presence of significant proportion of overweight/obesity

among severely ill psychiatric patients. Based on these findings the following points are

forwarded to the stake holders.

**For health professionals**

- Psychiatric patients should be properly educated on the impact of overweight/obesity during follow up visit in health facility or at household levels by health extension workers.
- Education should focus on positive health behaviors such as regular physical exercise, routine screening for overweight/obesity to maximize quality of life.
- Therefore, is also a need for health education to patients with psychiatric disorders about lifestyle modification through exercise, and ceasing alcohol consumption as preventative measures for overweight/obesity

**For public health sector**

- Integrated psychiatric treatment and medical screening service should be implemented at Hospital psychiatric center, to reduce missed opportunity.

**For researchers**

- Further prospective study should be conducted to identify the biological mechanism of psychiatric disorders for the development of overweight/obesity or to establish cause-effect relationship between the development of overweight/obesity and psychiatric illness.

# **7. References**

1. WHO. World Obesity Day: Understanding the social consequences of obesity. 2017;10(10).

2. World Health Organization. Global status report on noncommunicable diseases. World Heal Organ. 2010;53(9):1689–99.

3. Ajayi IO, Adebamowo C, Adami H-O, et al. Urban–rural and geographic differences in overweight and obesity in four sub-Saharan African adult populations: a multi-country cross-sectional study. *BMC Public Health*. 2016;16(1):1126.

4. Survey H. Ethiopia Demographic and Health Survey 2016,Central Statistical Agency Addis Ababa, Ethiopia. 2017.

5. Jan Pringle, Yvonne Laird DDS. Obesity and Mental Health:The Scottish Collaboration for Public Health Research and Policy. Univ Edinburgh. 2019;(June):6–68.

6. WHO. World Health Organization report about worlwide impact of Obesity and overweight. 2016;

7. Prevention(CDC) CFDC. Burden of Overweight and Obesity among adult populations. 2017;125–6.

8. Marcus MD. Obesity : Is it a Mental Disorder ? j life long learning in psychiatric . 2014;12(4):476–88.

9. Wilson AL GG. Overweight or obese young people are not at increased risk of depression but young people with depression are at increased risk of obesity. Diabetes Metab Syndr Clin Res Rev. 2014;17:112.

10. Aldossari KK, Shubair MM, Al-ghamdi S, Al-zahrani J, Alajmi M, Mastour S, et al. The association between overweight / obesity and psychiatric disorders : A population based cross-sectional study in Saudi Arabia. Saudi J Biol Sci. 2021;28(5):2783–8.

11. Nahar S, Al-Qahtani AA A-AS eta al. Association between obesity and mental disorders among male students of King Khalid University, Abha, Saudi Arabia. Saudi J Obes. 2015;(3):48–54.

12. Kamel A, Abuhegazy H, Ismail A, Sherra K, Ramadan M, Mekky A, et al. The prevalence of obesity in a sample of Egyptian psychiatric patients. Egypt J Psychiatr. 2016;37(June):157–65.

13. Wioletta Żukiewicz-Sobczak PW et al. Obesity and poverty paradox in developed countries. Ann Agric Env Med. 2014;21(3):590–594.

14. Association AP. Diagnostic and Statistical Manual of Mental Disorders FIFTH EDITION (DSM_5). Encyclopedia of Applied Psychology, Three-Volume Set. 2004. 607–614 p.

15. Jaykaran Charan and Tamoghna Biswas. How to calculate sample size for different study design in medical research? Indian J Psychol Med. 2013;35(2):121–6.

16. Sintayehu Asaye SB. Metabolic syndrome and associated factors among psychiatric patients in Jimma University Specialized Hospital, South West Ethiopia,Clin Res Rev. 2018. p. 753–60.

17. Gelaye B, Girma B, Lemma S, Berhane Y. Prevalence of Metabolic Syndrome among Working Adults in Ethiopia Prevalence of Metabolic Syndrome among Working Adults in Ethiopia. Int J Hypertens. 2011;(10).

18. Cerezo C, Segura J PM et al. Guidelines updates in the treatment of obesity or metabolic syndrome and hypertension. Curr Hypertens Rep. 2013;(15):196–203.

19. WHO. WHO Guidelines on physical activity and sedentary behaviour for children and adolescents , adults and older adults for consultation only. 2020;(March):1–34.

20. Ryan H, Trosclair A, Gfroerer J. Adult Current Smoking: Differences in Definitions and Prevalence Estimates the National Health Interview Survey (NHIS) and the National Survey on Drug Use and Health (NSDUH).USA Methods. 2008. J ofEnvironmental Public Heal. 2012;2012:11.

21. Getachew T, Defar A, Teklie H, Gonfa G, Bekele A, Bekele A. Magnitude and predictors of excessive alcohol use in Ethiopia : Findings from the 2015 national non- communicable diseases STEPS survey. EthiopJ Heal Dev. 2017;(31):312–9.

22. Annamalai A, Kosir U, Tek C. Prevalence of obesity and diabetes in patients with schizophrenia. World J Diabetes. 2017;8(8):390–6.

23. Mekonnen T, Animaw W, Seyum Y. Overweight / obesity among adults in North- Western Ethiopia : a community-based cross sectional study. BMC Obes. 2019;6(8):1–6.

24. Care PH, Micanti F, Pecoraro G, Mosca P, Riccio F, Galletta D. Primary Health Care : Open Access Obesity and Psychiatric Disorders in a Sample of Obese Candidates for Bariatric Surgery in Campania Region. Prim Heal Care. 2017;7(1):1–7.

25. Chouinard V, Pingali SM, Chouinard G, Henderson DC, Mallya SG, Cypess AM, et al. Factors associated with overweight and obesity in schizophrenia , schizoaffective and bipolar disorders. Psychiatry Res. 2016;11:1–7.

26. Weiss F, Barbuti M, Carignani G, Calderone A, Santini F, Maremmani I, et al. Psychiatric Aspects of Obesity : A Narrative Review of Pathophysiology and Psychopathology. J Clin Med. 2020;(9):1–18.

27. Weschenfelder J, Bentley J, Himmerich H, Bentley J, Himmerich H, Weschenfelder J. Physical and Mental Health Consequences of Obesity in Physical Women and Mental Health Consequences of Obesity in Women. Licens IntechOpen. 2018;(3):17–9.

28. Chao AM, Wadden TA, Berkowitz RI. Obesity in Adolescents with Psychiatric Disorders. 2019;21.

29. Husky MM, Mazure CM, Ruffault A. Differential Associations Between Excess Body Weight and Psychiatric Disorders in Men and Women Downloaded. J WOMEN’S Heal. 2017;00(00).

30. Hughes A, Kumari M. Unemployment , underweight , and obesity : Findings from Understanding Society ( UKHLS ). Prev Med (Baltim). 2017;97:19–25.

# **8. Annex**

**Dire Dawa University**

**College of Medicine and Health Sciences**

PARTICIPANT INFORMATION SHEET AND INFORMED VOLUNTARY CONSENT FORM

My name is (_____________________). I am working as a data collector for the study being conducted in this institution by **Dilnessa Fentie** and his co-researchers who are working at Dire Dawa University, the College of Medicine and Health Sciences. I kindly request you to lend me your attention to explain you about the study and being selected as the study participant.

1. The study/project title: overweight/obesity and associated factors patient with psychiatric disorder in Dire Dawa, East Ethiopia, 2021**.**

2. Purpose/Aim of the study: The aim of the study is to investigate the burden of overweight/obesity and associated factors patient with psychiatric disorder in Dire Dawa, East Ethiopia, 2021. The findings of this study can be of a paramount importance for health policy makers, researchers and for patients who are suffering from these disorders. If the syndrome exists to consult the patient and the care provider on taking appropriate measurement (life style modification, physical activity and taking medication).

3. Procedure and duration: I will give you an interviewer administer questionnaires concerning this study to provide me with pertinent data that is helpful for the study, so I kindly request you to spare me this time for filling the questionnaire.

4. Risks and benefits: The risk of being participating in this study is very minimal, but only taking few minutes from your time. There would not be any direct payment for participating in this study. But the findings from this research may reveal important information for the community.

5. Confidentiality: The information you will provide us will be confidential. There will be no information that will identify you in particular. The findings of the study will be general for the study community and will not reflect anything particular of individual persons. The questionnaire will be coded to exclude showing names. No reference will be made in oral or written reports that could link participants to the research.

6. Rights: Participation for this study is fully voluntary. You have the right to declare to participate or not in this study. If you decide to participate, you have the right to withdraw from the study at any time and this will not label you for any loss of benefits which you otherwise are entitled. You do not have to answer any question that you do not want to answer.

7. Contact address: If there are any questions or enquires you may have about the study or the procedures, please contact: the principal investigator by +251 913376812 or the Institutional Research and Ethics Review Committee (RERC) office phone 0254115427, Dire Dawa, Ethiopia.

8. Declaration of informed voluntary consent: I have read/ was read to me the participant information sheet. I have clearly understood the purpose of the research, the procedures, the risks and benefits, issues of confidentiality, the rights of participating and the contact address for any queries. I have been given the opportunity to ask questions for things that may have been unclear. I was informed that I have the right to withdraw from the study at any time or not to answer any question that I do not want. Therefore, I declare my voluntary consent to participate in this study with my initials (signature).

Name and signature of participant: ______________________ ___________ Date _________

Name and signature of Data Collector: ___________________ ___________ Date _________

N.B

- This is signed face to face in the presence of the data collector.
- Please provide a copy of this signed consent to the participant, and Keep copy for PI.

INFORMATION SHEET AND INFORMED VOLUNTARY CONSENT FORM

FOR HEAD OF INSTITUTION (UNIVERSITY)

My name is (_____________). I am working as a data collector for the study being conducted in this Institution by **Dilnessa Fentie** and his co-researchers who are working at Dire Dawa University, the College of Medicine and Health Sciences. I kindly request you to lend me your attention to explain you about the study and your institution being selected as the study setting.

1. The study/project title: Metabolic syndrome and associated factors patient with psychiatric disorder in Dire Dawa DCRH, East Ethiopia, 2021

2. Purpose/Aim of the study: The aim of the study is to investigate the burden of Metabolic syndrome and associated factors patient with psychiatric disorder in Dire Dawa DCRH, East Ethiopia, 2021. The findings of this study can be of a paramount importance for health policy makers, researchers and for patients who are suffering from visual disorders. If the syndrome exists to consult the patient and the care provider on taking appropriate measurement (life style modification, physical activity and taking medication).

3. Procedure and duration: I will give you 24 an interviewer administers questionnaires and 5ml of venous blood will be taken for laboratory tests concerning this study to provide me with pertinent data that is helpful for the study. The questionnaire will take about 25 minutes, so I kindly request you to spare me this time for filling the questionnaire.

4. Risks and benefits: The risk of being participating in this study is very minimal, but only taking few minutes from participants’ time. There would not be any direct payment for participating in this study. But the findings from this research may reveal important information for the community.

5. Confidentiality: The information participants will provide us will be confidential. There will be no information that will identify participants in particular. The findings of the study will be general for the study community and will not reflect anything particular of individual persons. The questionnaire will be coded to exclude showing names. No reference will be made in oral or written reports that could link participants to the research.

6. Rights: Participation for this study is fully voluntary. The participants have the right to declare to participate or not in this study. If they decide to participate, they have the right to withdraw from the study at any time and this will not label them for any loss of benefits which they otherwise are entitled. They do not have to answer any question that they do not want to answer.

7. Contact address: If there are any questions or enquires you may have about the study or the procedures, please contact: the principal investigator by +251 913376812 or the Institutional Research and Ethics Review Committee (RERC) office phone 0254115425, Dire Dawa, Ethiopia.

8. Declaration of informed voluntary consent:

I have read the participant information sheet. I have clearly understood the purpose of the research, the procedures, the risks and benefits, issues of confidentiality, the rights of participating and the contact address for any queries. I have been given the opportunity to ask questions for things that may have been unclear. I was informed that participants have the right to withdraw from the study at any time or not to answer any question that they do not want. I am also informed that the Institution has the right to stop this study from being conducted if any misdeeds and unethical procedures are observed during the data collection process in the Institution’s premises. Therefore, I declare my voluntary consent on behalf of the institution management to allow this study to be conducted here in the institution with my initials (signature).

Name and Signature of Head of the Hospital: ___________________ ________ Date ________

Name and Signature of Data Collector: ________________________ ________ Date ________

N.B

- This is signed face to face in the presence of the data collector.
- Please provide a copy of this signed consent to the responsible head, and keep copy for PI.
- Keep copy of signed consent to Principal investigator, which may be asked if need arises.

| Annex-iii. English versions of questionnaires | |
| --- | --- |
| **Code -------------------------------------**  **Types of Study group-----------------------** |  |
| **1. Socio-demographic data** |  |
| 1. Sex | 1. Female  2. Male |
| 1. If the respondent is female ask about obstetrics history (pregnancy status, history of contraceptive use for the past one year) | --------------------------  -------------------------- |
| 1. Age of the respondent: | **-------------------** |
| 4. Resident: | 1. Urban  2 .Rural |
| 5. Educational status | 1. Illiterate  2. Read and Write  3. Primary Education 5. Secondary Education  6. College and Above |
| 6. Occupation: | 1. Housewife  2. Governmental  3. Non-governmental 4. Private  5. Student |
| 7. Marital status: | 1. Married 2. . Single 3. Divorced 4. Widowed |
| 8. Family history of medical illness | 1 Hypertension  2. Cardiac illness  3. Diabetes  4 others, specify_______ |
| 2. **Clinical data** |  |
| 1. Diagnosis category of Psychiatric disorder (types of psychiatric disorder) | 1.Schizophrenia  2.Schizoaffective Disorder  3.Major Depressive  4. Bipolar Disorder |
| 2. Duration of illness(years/months) |  |
| 3.Anti-psychotic class | 1.Typical  2.Atypical  3.mood stabilizer  4. Antidepressant |
| 4.Duration of the current psychiatric medication she/ he is on__________ | 1 <5  2 5-10  3 >10 |
| 5. Additional psychiatric medication if any | Name______________ duration---------------- |
| 6.Are you currently receiving any drug (medication) other than anti  Psychiatric prescribed by a doctor or other health worker? | 1. Yes 2. No 3. If yes name of the drug------------- |
| 1. **Behavioral measurement** |  |
| 1. Do you ever smoke any tobacco products, such as cigarettes,   cigars or pipes? | 1. Yes  2. No  3.If the answer is no, go to question no 5 |
| 1. Do you currently smoke any tobacco products, such as cigarettes, cigars or pipes? | 1.yes  2.No |
| 1. Do you remember how long ago it was? In years or in months or in weeks | 1.<one year  2.≥one year |
| 1. On average, how many times do you smoke each day? |  |
| 5.Have you ever consumed an alcoholic drink such as tela, tej,  areke, beer, wine, whisky……… | 1. Yes  2. No  3. If no, go to question no 9 |
| 1. Do you currently drink any alcohol products(past 30 days)? | 1.yes  2.No |
| 1. Past alcoholic drinker (drank in the past 12 months) | 1.yes  2.No |
| 8. How frequently have you had at least one alcoholic drink? | 1. Daily  2. 5-6 days per week  3. 1-4 days per week  4. 1-3 days per month  5. Less than once a  month |
| 8.Does your work involve vigorous-intensity activity that causes large increases in breathing or heart rate like (carrying or lifting heavy loads, digging or construction work) for at least 10 minutes continuously?  If the answer is yes how much time do you spend doing vigorous-intensity activities at work on  a typical day? ------  If the answer is no, go to question no 9 | 1. Yes  2. No |
| 1. In a typical week, on how many days do you do vigorous-   intensity activities as part of your work? number of days |  |
| 1. Does your work involve moderate-intensity activity that causes small increases in breathing or heart rate such as brisk walking (or carrying light loads) for at least 150 minutes/week? | 1. Yes 2. No |
| 1. In a typical week, on how many days do you do moderate-   intensity activities as part of your work? number of days |  |
| 4. **Physical Measurements** |  |
| 1 Height in cm |  |
| 2 Weight in kg |  |
| 3 BMI |  |

**የተሳታፊዎች መረጃ ቅፅ**

**ድሬዳዋ ዩኒቨርሲቲ የጤና ሳይንስ እና ሕክምና ኮሌጅ-ሜዲካል ትምህርት ክፍል**

የተሳትፎ መረጃ ወረቀት እና በመረጃ የተደገፈ የፈቃደኝነት ቅፅ

ስሜ (_____________________)ነው፡፡ በድሬዳዋ ዩኒቨርሲቲ ሕክምና እና የጤና ሳይንስ ኮሌጅ በሚሠሩ በድልነሳ ፈንቴ እና ባልደረቦቻቸው አማካይነት ለሚካሄደው ጥናት እንደ መረጃ ሰብሳቢነት ሆኘ እየሠራሁ ነው ፡፡ እርሶም በዚህ ጥናት ላይ እንዲሳተፉ ተጋብዘዋል፡፡

1. ጥናቱ / የፕሮጀክቱ ርዕስ-ሜታብሊክ ሲንድሮም እና አጋላጭ ነገሮች በከባድ የአእምሮ ህመምተኞች ላይ
2. የዚህ ጥናት ግኝቶች ለጤና ፖሊሲ አውጪዎች ፣ ተመራማሪዎች እና በሜታብሊካል ሲንድሮም ለሚሰቃዩ ህመምተኞች ከፍተኛ ጠቀሜታ ይኖረዋል፡፡ ለህክምና ሰጪ አካላትም ተገቢውን እርምጃ እንዲወስዱ ማለትም ማማከር፣ የኑሮ ዘዴን ማስተካከል፣ የአካል ብቃት እንቅስቃሴ መስራት እንዲሁም ተገቢውን መድሐኒት እንዲወስዱማድረግ፡፡
3. የአሠራር ሂደት እና የቆይታ ጊዜ: - ጥናቱን የምናደርገው በቃለምልልስ እና ላቦራቶሪ ምርመራዎችን በማድረግ ይሆናል፡፡ቃለመጠይቁ ወደ 25 ደቂቃ የሚወስድ ሲሆን የደም ናሙና ደግሞ 5 ደቂቃዎችን ይወስዳል
4. አደጋዎች እና ጥቅሞች-በዚህ ጥናት ውስጥ የመሳተፍ አደጋ በጣም አናሳ ነው ፣ ግን ከእርስዎ ጊዜ ጥቂት ደቂቃዎችን ብቻ ይወስዳል ፡፡ በዚህ ጥናት ውስጥ ለመሳተፍ ቀጥተኛ ክፍያ አይኖርም ፡፡ ነገር ግን ከዚህ ምርምር የተገኘው ውጤት ለማህበረሰቡ ጠቃሚ መረጃዎችን ሊያሳይ ይችላል ፡፡
5. ሚስጥራዊነት-እርስዎ የሚሰጡን መረጃ ሚስጥራዊ ይሆናል ፡፡ በተለይ እርስዎን የሚለይ መረጃ አይኖርም ፡፡ የጥናቱ ግኝቶች ለጥናቱ ማህበረሰብ አጠቃላይ ይሆናሉ እንጂ የግለሰቦችን ማንነት የሚያንፀባርቅ አይሆንም ፡፡ መጠይቁ ስሞችን ከማሳየት እንዲቆጠብ ኮድ ይደረጋል ፡፡ ተሳታፊዎችን ከምርምሩ ጋር ሊያገናኝ የሚችል በቃል ወይም በፅሁፍ ሪፖርቶች ውስጥ ማጣቀሻ አይሰጥም
6. መብቶች-የዚህ ጥናት ተሳትፎ ሙሉ በሙሉ በፈቃደኝነት የሚደረግ ነው ፡፡ በዚህ ጥናት ውስጥ ለመሳተፍ ወይም ላለመሳተፍ የማወጅ መብት አለዎት ፡፡ ለመሳተፍ ከወሰኑ በማንኛውም ጊዜ ከጥናቱ የመውጣት መብት አለዎት ፡፡ መመለስ የማይፈልጉትን ማንኛውንም ጥያቄ መመለስ የለብዎትም ፡፡
7. የአድራሻ አድራሻ-ስለ ጥናቱ ወይም ስለ አሠራሩ የሚጠይቋቸው ጥያቄዎች ካሉ እባክዎ በዋና ተመራማሪው በ 251 913376812 ወይም በተቋሙ ጥናትና ሥነ ምግባር ግምገማ ኮሚቴ (RERC) ቢሮ ስልክ 0254115425 ያነጋግሩ፡፡

**የፈቃደኝነት ፎርም**

የተሳታፊውን የመረጃ ወረቀት አንብቤዋለሁ/ ተነቦልኛል፡፡ የምርምሩን ዓላማ ፣ አሰራሮችን ፣ አደጋዎችን እና ጥቅሞችን ፣ ሚስጥራዊነትን ጉዳዮች ፣ የተሳትፎ መብቶችን እና ለማንኛውም ጥያቄዎች የግንኙነት አድራሻ በሚገባ ተረድቻለሁ ፡፡ ግልጽ ባልሆኑ ጉዳዮች ላይ ጥያቄዎችን መጠየቅ እድሉ ተሰጥቶኛል ፡፡ በማንኛውም ጊዜ ተሳትፎየን የማቁረጥ ወይም ያንን ጥያቄ ላለመመለስ መብት እንዳለኝ ተገለፀልኛል

የተሳታፊ ፊርማ/አሻራ፥-------------------ቀን______

የመረጃ አሰባሳቢው ስም _______________ ፊርማ __________ ቀን______

**Amharic Version of the Questionnaire**

|  | 1) ማንነትን የሚመለከት ጥያቄዎች |  |
| --- | --- | --- |
| 1.1 | ፆታ | 1. ወንድ  2. ሴት  ተጠሪ ሴት ከሆኑ  ስለ የወሊድ ታሪክ (የእርግዝና ሁኔታ ፣ ላለፈው አንድ ዓመት የእርግዝና መከላከያ አጠቃቀም ታሪክ)  ---------------------------  መልስዎ በአሁኑ ጊዜ እርጉዝ ከሆኑ ወይም የእርግዝና መከላከያ ተጠቃሚ ከሆኑ የጥናቱ አካል አይሆኑም |
| 1.2 | ዕድሜ | --------------- |
| 1.3 | የጋብቻ ሁኔታ | 1. በትዳር ላይ ያሉ  2. ያላገባ/ች  3. የፈታ/ች  4. በሞት የተለየ |
| 1.4 | የትምህርት ደረጃ | 1. አልተማርኩም  2. ማንብብ መፃፍ እችላላሁ  3. አንደኛ ደረጃ ያጠናከኩ  4. ሁለተኛ ደረጃ ያጠናከኩ  5. ኮሌጅ እና ከዚያበላይ  6. ሌላ ካለ |
| 1.6 | ስራ | 1. የቤት እመቤት  2. የመንግስት ሠራተኛ  3. መንግስታዊ ያልሆነ ድርጅት ሠራተኛ  4. ግል |
| 1.7 | የመኖሪያ ቦታ | 1. ገጠር  2. ከተማ |
| 1.8 | የወር ገቢ | -------------------ብር |
|  | **2) ባህሪን የሚመለከቱ ጥያቄዎች** |  |
|  | የሲጃራ አጠቃቀምን የሚመለከቱ ጥያቄዎች |  |
| 2.1 | ሲጃራና የሲጃራ ውጤቶች አጭሰው ያውቃሉ? | 1. አዎ  2. የለም  መልሶዎ የለም ከሆነ ወደ ጥያቄ ቁጥር 2.5 ይሂዱ |
| 2.2 | በአሁኑ ጊዜ ሲጃራና የሲጃራ ውጤቶችን ይጠቀማሉ? | 1. አዎ  2. የለም |
| 2.3 | ማጨስ ከጀመሩ ምን ያህል ጊዜ ሆነዎት? | በዓመት--------------- ወይም  በወር-------------------- ወይም  በሳምንት-------------------- |
| 2.4 | በአማካኝ በቀን ምን ያህል ጊዜ ያጨሳሉ? | ------------------------ |
|  | **የአልኮል አጠቃቀምን የሚመለከቱ ጥያቄዎች** |  |
| 2.5 | የአልኮል መጠጥ እንደ ጠላ፣ ጠጅ፣ አረቄ፣ ቢራ፣ ወይን፣ ውስኪ…ወዘተ  ጠጥተው ያውቃሉ? | 1. አዎ  2. የለም  መልሶዎ የለም ከሆነ ወደ ጥያቄ ቁጥር 2.9 ይሂዱ |
| 2.6 | በአሁኑ ጊዜ አልኮል መጠጥ ይጠቀማሉ(ባለፈው 30 ቀናት ውስጥ)? | 1. አዎ  2. የለም |
| 2.7 | ባለፈው አንድ አመት ውስጥ ል መጠጥ ተጠቀመዋል? | 1. አዎ  2. የለም |
| 2.8 | ምን ያህል አዘውትረው የአልኮል መጠጥ ይጠቀማሉ? | 1. በየቀኑ  2. ከ5 – 6 ቀን በሳምንት  3. ከ1 – 4 ቀን በሳምንት  4. ከ1 – 3 ቀን በወር  5. በወር ውስጥ ከ1 ባነሰ መጠን |
|  | **አካል ብቃት እንቅስቃሴን የሚመለከት ጥያቄ** |  |
| 2.9 | የሚሰሩት ስራ ከባድ የአካል እንቅስቃሴዎችን ማለትም የመተንፈስ አቅምንና  የልብ ምትን የሚጨምሩ ስራዎችን ያጠቃልላል? (ቢያንስ ለተከታታይ 10  ደቂቃዎች) ለምሳሌ፡- ከባድ ዕቃዎችን መሸከም መቆፈር …. | 1. አዎ  2. የለም  መልሶዎ አዎ ከሆነ በቀን ውስጥ ለስንት ሰዓት?  -----------------------------------------  መልሶዎ አይ ከሆነ ወደ ጥያቄ ቁጥር 2.10 ይሂዱ |
| 2.10 | በሳምንት ውስጥ ለምን ያህል ቀን ከባድ እንቅስቃሴዎችን በስራዎት ውስጥ  ያከናውናሉ? | ----------------------------------- |
| 2.11 | የሚሰሩት ስራ መካከለኛ የአካል እንቅስቃሴዎችን ማለትም ትንሽ የመተንፈስ  አቅምንና የልብ ምትን የሚጨምሩ ስራዎችን ያጠቃልላል? (ቢያንስ  ለተከታታይ 10 ደቂቃዎች) ለምሳሌ፡- ቀላል ዕቃዎችን መሸከም | 1. አዎ  2. የለም  መልሶዎ አዎ ከሆነ በቀን ውስጥ ለስንት ሰዓት?  ------------------------------------  መልሶዎ አይ ከሆነ ወደ ጥያቄ ቁጥር 2.12 ይሂዱ |
| 2.10 | በሳምንት ውስጥ ለምን ያህል ቀን መካከለኛ እንቅስቃሴዎችን በስራዎት  ውስጥ ያከናውናሉ? | ------------------------------ |
| 2.11 | ከቦታ ቦታ ለመንቀሳቀስ የእግር ጉዞ ቢያንስ ለተከታታይ 10 ደቂቃዎች  ይጠቀማሉ? | 1. አዎ  2.የለም  መልሶዎ አዎ ከሆነ በቀን ውስጥ ለስንት ሰዓት?  -------------------------------------  መልሶዎ አይ ከሆነ ወደ ጥያቄ ቁጥር 3.1 ይሂዱ |
| 2.12 | በሳምንት ውስጥ ለምን ያህል ቀን የእግር ጉዞ ለመንቀሳቀስ ይጠቀማሉ? |  |
|  | **3 ክሊኒካል መረጃ** |  |
| 3.1 | የአእምሮ በሽታ (የአእምሮ በሽታ ዓይነቶች)  የህመም ጊዜ (ዱረሽን)---------------- | 1. ስንዞፊረኒያ 2. ድብረሽን 3. ባይቦላር ዲስወርደር 4. ሲንዞ አፈክቲብ ዲስወርደር |
| 3.2 | የቤተሰብ በሽታ ታሪክ | 1 የደም ግፊት  2. የልብ ህመም  3. የስኳር በሽታ  4 ሌሎች ፣ ይጥቀሱ---------- |
| 3.3 | የአእምሮ ህክምና መድሃኒት ስም እና የመድኃኒት መጠን___ |  |
|  | 4) የአካል መጠን መለከኪያ |  |
| 4.1 | ቁመት | በሳ.ሜትር---------------- |
| 4.2 | ክብደት | በኪ.ግራም-------------- |
| 4.3 | የሰውነት ክብደት መጠን(BMI) | ----------------- |
