# Supplementary material for: Prevalence and associated factors of overweight/obesity among severely ill psychiatric patients in Eastern Ethiopia: A comparative cross-sectional study
Source: PLoS One. 2022 Mar 2;17(3):e0264461. doi: 10.1371/journal.pone.0264461 (PMC8890638; doi:10.1371/journal.pone.0264461)
Supplement: Attachment — Submitted filename: renamed_e168b.docx [file pone.0264461.s003.docx]

Date: November 11/11/2021

**Bottom of Form**

**To: Journal of PLOS ONE, editors-in-chief**

**Subject:** Submitting response to reviewers' comments for manuscript number: [PONE-D-21-28472] - [EMID: f24d0812985cdb44]

PONE-D-21-28472

Dear Dr. Aleksandra Barac,

Thank you for giving us the opportunity to submit a revised draft of the manuscript “**Prevalence and Associated Factors of Overweight/Obesity among severely ill Psychiatric Patients in Eastern Ethiopia**” for publication in the *Journal of PLOS ONE.* We appreciate the time and effort that you and the reviewers dedicated to providing feedback on our manuscript and are grateful for the insightful comments and valuable improvements to our paper. We have incorporated most of the suggestions made by the reviewers and those changes are highlighted within the manuscript. Please see below, in green color, for a point-by-point response to the reviewers’ comments and concerns. All changes stated here in response to reviewers’ comments and concerns are incorporated in the revised version of the manuscript. All page and line numbers refer to the revised manuscript file with tracked changes. We look forward to hearing from you in due time regarding our submission and to respond to any further questions and comments you may have.

With Kind regards,

Comments from Reviewer 1

The overall research hypothesis and how the research question were technically sound and interesting to share with the readers, however the way the data were presented had major problems. Numerous mistakes or inaccuracies with language made some parts of the manuscript difficult to read or follow the idea.

**The response of the authors**: We thank the reviewer for the comment. We have identified the problem and made the necessary corrections throughout the revised manuscript.

Examples include:

1. Line 36 : It's ninety six, sex and age matched. Such a mistake in the abstract is very confusing to the reader.

**The response of the authors:** We thank the reviewer for the comment and sorry for vague statements. Correction has made in the revised manuscript (page 2, line no.35-36).

2. Line 46: It's weight reduction not treatment, since the scope of your paper is around obesity. The term wight treatment might refer to treating underweight as well.

**The response of the authors**: The reviewer is correct, it is out of our scope and we have modified the contents in the revised manuscript (page 3, line no.48-49)-

Other problems with literature review or methodology that had missing important information for example:

3. Line 51: I'd rather see statistics from Africa not Europe, since your research is done in Ethiopia. Same data can be obtained from WHO statistics too.

The response of the authors: We thank the reviewer for the suggestions and concern. We have added references indicate about African and Ethiopia to the manuscript on (page 3, line no. 54-56).

4. Line 73: Which sociodemographic characteristics exactly? the was it's written in your review is vague

The response of the authors: We would like to appreciate the reviewer’s comment and concern. We have modified the suggested content to the revised manuscript on (page 4, line no. 87-88).

5. Line 91: There is nothing called non=psychiatric individuals. It could be normal controls not suffering from psychiatric illnesses for example.

**The response of the authors:** We thank the reviewer for the comment. We have identified the problem and made the necessary corrections in the revised manuscript (page 5, line no.102-105).

6. Line 97: Which edition of the DSM? Were the diagnoses made by specialist psychiatrists or GPs and family physicians?

**The response of the authors:** The reviewer is correct, and we have incorporated the contents into the revised manuscript (page 5, line no. 100-102). DSM-5 and psychiatrists specialist

7. Line 101: Why were those populations specifically excluded from the study?

**Response of the authors:** We would like to appreciate the reviewer’s comment and concern. **We exclude those who were pregnant and physically deformed** study participants because **Body Mass Index (BMI)** was measured by dividing a person’s weight in kilograms by his or her height in meters squared (kg/m^2^). The height of the participants was measured using a stadiometer with the participant standing upright with the heel, buttock, and upper back along the same vertical plane. The weight of the participants was measured using a calibrated weighing scale with the participant not wearing shoes and heavy clothes. We believe that physically deformed and pregnant individual measurements might result in abnormal BMI status, thus affecting the final outcomes.

I would suggest doing this revisions and also doing an English proof reading by more fluent

researcher or possibly whose native language is English and then resubmit.

**The response of the Authors:** We would like to thank you for your comment and we have edited the grammatical issues in the revised manuscript.

Reviewer #2: I hope you will find these comments to be helpful

The response of the Authors**: Thank you very much**

1) some paragraphs need references ; first paragraph of the abstract and introduction ; to make the facts more reliable

**The response of the authors:** The reviewer is correct, and we have incorporated the comments into the revised manuscript (page 3, line no.52). However, the first paragraph of the abstract was taken from (references 1,3,4)

2) the authors mentioned that their population was severely ill psychiatric patients, and again they mentioned they were selected from the outpatient! I find this to be contradicting. Do they mean chronic visitors?

**The response of the authors:** We appreciate the reviewer’s concern and are sorry for the unclarity. Severely ill psychiatric patients (=patients with a diagnosis of one or more of those psychiatric disorders such as schizophrenia, bipolar disorder, major depressive disorder, and schizoaffective disorders) and have chronic follow-up/chronic psychiatric outpatient visitors.

3) what are the criteria of severely ill?

**The response of the authors:** We would like to appreciate your concern. We were considered the severity of psychiatric disorders as described in DSM-5. Our criteria was based on the number of symptoms, level of distress caused by the intensity of symptoms, and impairment in social and occupational functioning.

4) Would severely ill patients be able to give a consent?

**The response of the authors:** We would like to appreciate your concern. We were selected severely psychiatric patients (schizophrenia, bipolar disorder, major depressive disorder, and schizoaffective disorders) and who attended the outpatient psychiatric clinic. Severely ill Clients who were unstable or unable to consent due to acute symptoms (as determined by the attending physician) were excluded. We were considered only stable study participants (those able to give consent).

5) the manuscript needs language editing

**The response of the Authors:** We would like to thank you for your constructive comments and concerns for forwarding them to us to enrich our paper. We have corrected all the comments and we have also edited the language in the revised manuscript
